# Keeping your options open: insights from Dppa2/4 into how epigenetic priming factors promote cell plasticity

**DOI:** 10.1042/BST20200873

**Published:** 2020-12-18

**Authors:** Mélanie A. Eckersley-Maslin

**Affiliations:** 1 Epigenetics Programme, Babraham Institute, Cambridge CB22 3AT, U.K.; 2 Peter MacCallum Cancer Centre, Melbourne, Victoria 3000, Australia; 3 Sir Peter MacCallum Department of Oncology, The University of Melbourne, Parkville, Victoria 3010, Australia

**Keywords:** cell fate, chromatin, Dppa2/4, epigenetic priming, epigenetics, plasticity

## Abstract

The concept of cellular plasticity is particularly apt in early embryonic development, where there is a tug-of-war between the stability and flexibility of cell identity. This balance is controlled in part through epigenetic mechanisms. Epigenetic plasticity dictates how malleable cells are to change by adjusting the potential to initiate new transcriptional programmes. The higher the plasticity of a cell, the more readily it can adapt and change its identity in response to external stimuli such as differentiation cues. Epigenetic plasticity is regulated in part through the action of epigenetic priming factors which establish this permissive epigenetic landscape at genomic regulatory elements to enable future transcriptional changes. Recent studies on the DNA binding proteins *Developmental Pluripotency Associated 2* and *4* (Dppa2/4) support their roles as epigenetic priming factors in facilitating cell fate transitions. Here, using Dppa2/4 as a case study, the concept of epigenetic plasticity and molecular mechanism of epigenetic priming factors will be explored. Understanding how epigenetic priming factors function is key not only to improve our understanding of the tight control of development, but also to give insights into how this goes awry in diseases of cell identity, such as cancer.

## Introduction

Mammalian development starts with a single totipotent zygote, capable of giving rise to all embryonic and extraembryonic cell types. The potency of early embryonic cells decreases as the embryo develops and cell fates are specified. The first cell fate decision occurs by the blastocyst stage when embryonic versus extraembryonic cell fates have been made. Shortly after, the three germ layers are specified during gastrulation. This plasticity in development, or the ability of cells to adapt to external cues and adopt new functions or identities, is regulated in part by the epigenome. While all cells at all stages of embryonic development contain the same genetic information, the availability of this DNA content is tightly regulated through epigenetic mechanisms which direct gene expression programmes to provide cell identity while simultaneously priming cells for change.

**Figure 1.**
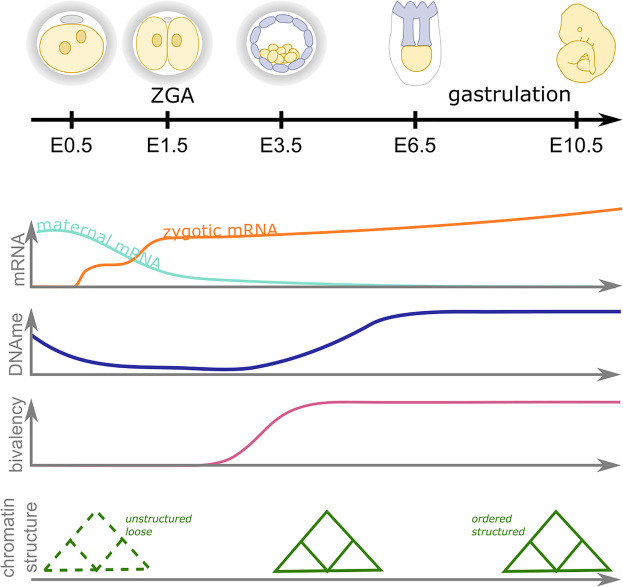
Epigenetic dynamics during early mammalian embryonic development. Zygotic genome activation (ZGA) occurs in two waves (orange line) in the late zygote and two cell stage (four to eight cell stage in humans) as maternal mRNA (aqua line) is degraded. Concurrently, there is a global wave of DNA demethylation and remethylation (blue line), an acquisition of chromatin bivalency (magenta line) as the higher order chromatin structure becomes more ordered (green). During gastrulation cell identities become more refined as the epigenome becomes more structured. Note embryo schematics are not to scale and timings are approximate and based on murine development.

The epigenome undergoes dramatic changes during the early stages of development (reviewed in [1–4]) (Figure 1). In mammals, DNA methylation is found predominantly on cytosine bases when followed by a guanine (CpG), and is generally associated with gene repression (reviewed in [5,6]). The majority of somatic cells, including the mature gametes, have high levels of CpG methylation. However, following fertilisation there is a global loss of DNA methylation which is only reinstated at the onset of gastrulation following implantation (Figure 1). Similarly, the chromatin landscape also changes dramatically during early development. Globally, the higher-order chromatin structure in zygotes is relaxed and loose, and becomes progressively more structured and ordered as development progresses [7,8]. At the level of the nucleosomes, the histones that make up chromatin are extensively post-translationally modified, and the pattern of acetyl, methyl, and other modifications reflects the accessibility and activity of the underlying genetic information. These histone modification patterns, while relatively stable in differentiated cells, undergo dynamic changes during early embryonic development (reviewed in [1,9]). It is thought that these global DNA methylation and chromatin dynamics resets the epigenetic landscape in early embryos, increasing cellular plasticity and facilitating the developmental potential of the cells.

By regulating gene expression, epigenetics has the potential not only to define current cell states but to shape how cells respond to external cues such as differentiation or stress. Epigenetic plasticity describes how malleable or flexible this regulation is: highly plastic cells have a wide range of cell identities available to them while non-plastic cells are static and unresponsive. By maintaining the chromatin in a state that has the potential to become either activated or repressed, epigenetic plasticity gives cells the flexibility to adopt different cell fates or functions. Similar to cell potency, epigenetic plasticity is a continuum of states and generally decreases as development progresses. How this epigenetic plasticity is regulated and its impact on embryonic development is only beginning to be understood. Here, the role epigenetic priming factors play in facilitating epigenetic plasticity will be explored, using the DNA binding proteins *Developmental Pluripotency Associated 2* and *4* (Dppa2/4) as exemplars of this emerging class of epigenetic regulators.

## What is an epigenetic priming factor and how could they work?

Epigenetic priming factors generate a permissive epigenetic landscape that facilitates future activation of gene expression programmes. This primed epigenetic landscape can take place at a range of genomic elements, including promoters and enhancers, and comes in many forms. Perhaps the best understood is the case of bivalent chromatin at promoters (reviewed in [10,11]). In pluripotent cells, this co-occurrence of both active associated H3K4me3 and repressive associated H3K27me3 modifications keeps developmental genes primed for future activation or silencing upon differentiation [12,13]. Promoters can also be primed by histone acetylation which is observed at ZGA-related promoters in Zebrafish where H3K27 acetylation marks promoters prior to their activation [14], and *Drosophila* where maternal H4K16 acetylation primes ZGA genes for future gene activation [15]. In addition to histone modifications, histone variants can also prime genes, which is observed in the case of the H2A variant H2A.Z(FV) which acts as a ‘placeholder’ at ZGA promoters in zebrafish embryos prior to their activation [16]. Priming also extends beyond promoters. Enhancers are frequently found inactive yet marked by H3K4me1 (primed enhancers) or the combination of H3K4me1, p300 and H3K27me3 (poised enhancers) [17,18]. These enhancer classes only acquire the ability to activate their target genes at later developmental stages (reviewed in [19]). In addition to chromatin features, poised RNA polymerase II [20] and/or antisense transcription [21] at epigenetically primed loci can help keep the locus permissive for future activation. This variety of diverse chromatin structures at a range of genomic features, including potentially many more still undiscovered, likely provide both specificity and redundancy in priming the underlying sequence for future activation. The identity and function of the priming factors required for the establishment and/or maintenance of these epigenetic landscapes is an ongoing area of research.

### Predicted features of epigenetic priming factors

**Figure 2.**
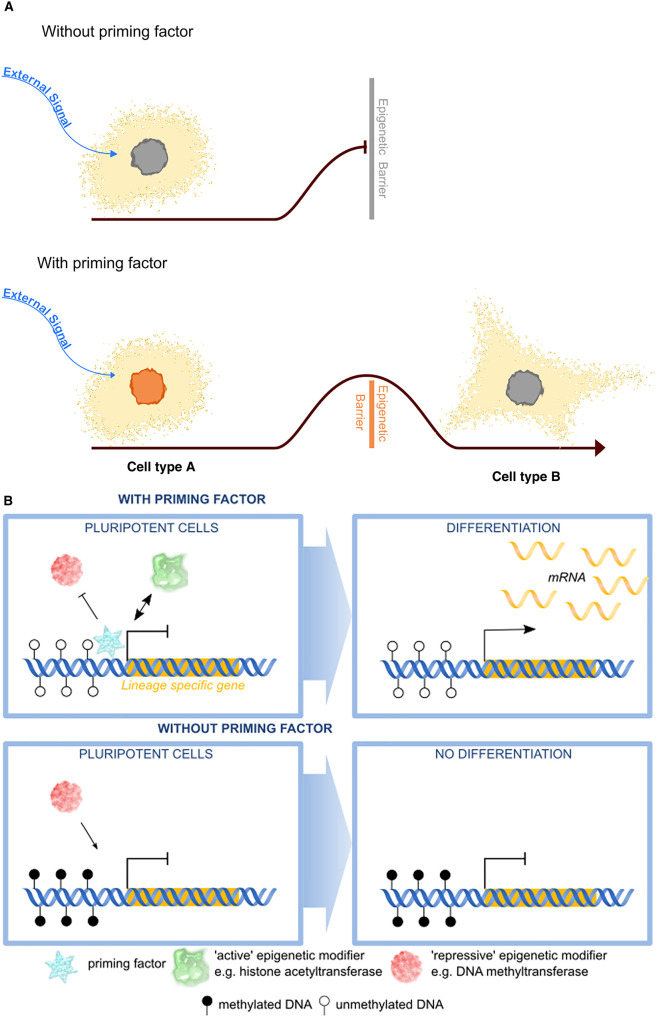
Mechanisms and significance of epigenetic priming. (**A**) In the absence of epigenetic priming, cells are unable to adopt new cell fates in response to external cues (bue arrow) due to high epigenetic barriers. However cells with epigenetic priming factors (orange nuclei) have a lowered epigenetic barrier and can more easily acquire new cell identities or functions in response to external signals (blue arrow). (**B**) Epigenetic priming factors (blue) may function at the molecular level at genomic regulatory elements of lineage specific genes (orange bar) by repelling DNA methylation (filled lollipops), preventing repressive epigenetic modifiers (red) from binding, or promoting active epigenetic modifiers (green) from binding. Upon differentiation these lineage specific genes can now be readily transcribed. In the absence of epigenetic priming factors (bottom panel), the epigenetic landscape is remodelled such that it is now no longer able to be activated efficiently upon differentiation.

There are several features of epigenetic priming factors that make them unique from other epigenetic and transcriptional regulators. One key feature of priming factors is the temporal uncoupling between the time they exert their molecular effects and resulting transcriptional and/or phenotypic consequences. For example, an epigenetic priming factor may prime gene regulatory elements in cell type A which are not required by that cell *per se* but are activated when the cell receives an external signal. Removing the epigenetic priming factor from cell type A would have minor phenotypic effects, however, this cell would now no longer be able to respond efficiently to the signal (Figure 2A). This has recently been elegantly shown in *C. elegans* where transient expression of TBX-37/38 primes the *lsy-6* gene for future activation 4 cell cycles later at a time when TBX37/38 is no longer expressed [21]. Additionally, in mammals priming factors bind and mark the albumin enhancer in precursor gut endoderm where it is inactive until hepatic specification later in development [22].

Epigenetic priming factors may have pioneer activity although this is not a requirement. Pioneer factors are able to bind DNA that is tightly packaged around a nucleosome thereby opening up otherwise inaccessible chromatin structures (reviewed in [23]). For example, the pioneer factor Zelda primes early zygotic enhancers in *Drosophila*, facilitating the subsequent binding of other factors during zygotic genome activation [24,25]. Moreover, in zebrafish, the pioneer factor Foxd3 recruits Brg1 to mediate chromatin priming and chromatin remodelling in early neural crest development [26]. In mouse ESCs, Foxd3 also has roles in priming enhancers, such as the *Alb1* enhancer, marking this enhancer for activation in later lineages [27]. However, while pioneering activity is obviously an advantage, it may not be a necessity in early development where the global epigenetic dynamics could enable factors without pioneering activity to bind their target sites.

Another important consideration is that initiating and maintaining an epigenetically primed structure are not necessarily performed by the same molecules. While epigenetic priming factors may be required to establish the primed state, this could be maintained and perpetuated in the absence of the initial trigger by the epigenetic machinery. For example, active genes can be maintained through positive feedback mechanisms between RNA polymerase II and trithorax group proteins [28] in the absence of the initiating transcription factor. Likewise, DNA methylation can be maintained through mitosis by the maintenance DNA methyltransferase Dnmt1, even if the *de novo* DNA methyltransferases Dnmt3a/3b are no longer being targeted to these sites. In this way, epigenetic priming factors do not need to continuously target their epigenetic state once they are established.

Epigenetic priming factors may have subtle roles in assisting cell fate transitions, acting as facilitators rather than gatekeepers of cell identity. By creating a permissive epigenetic environment at their targets, epigenetic priming factors may increase the frequency or temporal dynamics of target gene activation to ensure timely and efficient adaptation of transcriptional programmes. While key regulators are necessary for the acquisition of new transcriptional programmes, epigenetic priming factors function more as facilitators, preparing cells for change, and may not be absolutely required. As such, manipulating levels of priming factors may result in subtle or delayed phenotypes, rather than complete absence expected for essential regulators, as is seen in the case of Dppa2/4 (see below) [29,30]. It is also likely that epigenetic priming factors have additional roles beyond facilitating cell fate transitions, for example following stress or injury or in response to hormonal or circadian fluctuations.

### Possible mechanisms by which epigenetic priming factors may function

Conceptually, epigenetic priming can be thought of in terms of activation barriers which need to be overcome for a cell to adopt a new fate or function (Figure 2A). The epigenetic plasticity of the cell describes how high these barriers are. Epigenetic priming factors function by lowering these barriers, facilitating the cell to acquire new identities. At a molecular level, epigenetic priming factors could function in several ways (Figure 2B). By binding genomic features, they could prevent repressive DNA methylation from encroaching and silencing the locus. Priming factors could repel repressive epigenetic modifiers, such as H3K9 histone methyltransferases from binding, or recruit active epigenetic modifiers such as histone acetyltransferases. Alternatively, they may initiate antisense transcription that keeps the locus open. This may not directly lead to gene activation, although some low level of transcription may be observed, but generates a permissive epigenetic landscape to enable activation of these features upon differentiation. In the absence of the priming factor, the locus may adopt a closed structure and gain DNA methylation, repressive epigenetic modifiers may now bind, or active epigenetic modifiers may no longer be effectively recruited. Consequently, the locus is unable to be effectively activated upon differentiation, leading to aberrant gene expression programmes long after the epigenetic priming factor is present.

## Dppa2 and Dppa4 as epigenetic priming factors in development

**Figure 3.**
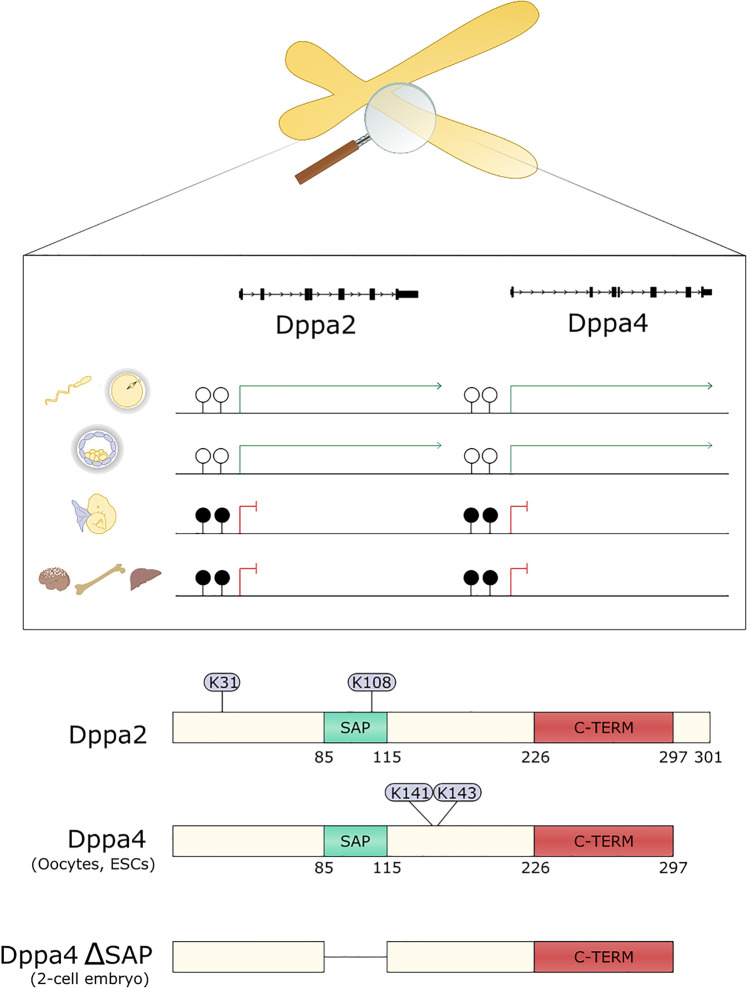
Developmental pluripotency associated 2 and 4. Top panel depicts the Dppa2/4 locus on mouse chromosome 16 or human chromosome 3. Dppa2/4 promoters are unmethylated in the germ line (top row) and preimplantation embryos (second row) where Dppa2/4 are expressed, and kept silent by promoter DNA methylation from E7.5 onwards (third row) and throughout the remainder of embryogenesis and in adult cells (bottom row). Bottom panel depicts the structure of Dppa2 and Dppa4 proteins showing SAP domain that binds DNA (green) and C-terminal domain (red) that binds histones. Residues sumoylated in ESCs are shown in circles. Numbers correspond to mouse Dppa2/4. Full length Dppa4 is found in most cell types while an alternatively spliced isoform found lacking the SAP domain is found in 2-cell embryos.

Emerging research has revealed that the small DNA binding proteins Dppa2/4 act as epigenetic priming factors and promote cell plasticity in early development. Originally identified in a screen for pluripotency factors [31], Dppa2/4 are expressed almost exclusively in early embryos (Figure 3). Somewhat perplexingly, zygotic knockout mice survive early embryogenesis only to succumb from lung and skeletal defects shortly after birth at a time that Dppa2/4 are no longer expressed [29,30]. This temporal uncoupling of the knockout mouse phenotype from when the proteins are present is one of the predicted features of epigenetic priming factors and led to predictions that epigenetic memories from earlier embryonic stages are important for proper development [30].

Dppa2/4 are arranged in tandem in a gene desert on mouse chromosome 16 or human chromosome 3 and, at least in mouse, are directly regulated by DNA methylation [32] (Figure 3). This keeps the genes silent following gastrulation through to adulthood, restricting their expression to early embryos and the germline when two global waves of DNA demethylation occur. These small ∼300 amino acid proteins lack any known enzymatic activity and instead have a SAP domain that binds DNA, a nuclear localisation signal and conserved C-terminal domain that associates with histones [29,33]. Dppa2/4 localise to euchromatin [30,33,34] with similar dynamics as histone H1 [33]. While they have a preference for GC rich promoter DNA, it is currently unknown how Dppa2/4 are targeted to specific genomic loci. In mouse, Dppa2/4 predominantly form heterodimers which is likely required for their function as manipulating levels of just one of the two proteins gives largely the same phenotype as mutating both [30,32]. Dppa2/4 are regulated post-translationally by SUMOylation [35,36] which inhibits their function. It remains to be uncovered whether additional post-translational modifications are involved in fine-tuning their activity.

### Priming zygotic genome activation

Activation of the embryonic genome following fertilisation represents a major developmental milestone as control is passed from the oocyte to the embryo proper. Zygotic genome activation takes place in two waves: a largely promiscuous minor wave followed by a major wave at the two-cell stage in mouse or four-to-eight-cell stage in humans (reviewed in [1]). Aspects of zygotic genome activation can be studied *in vitro* using serum-grown mouse embryonic stem cells which contain a rare subpopulation of 2C-like cells that share features of the two-cell embryo [37–40]. Using this model, several studies have revealed an essential role for Dppa2/4 in regulating 2C-like cells and the ZGA-associated transcripts, including the Zscan4 cluster, MERVL endogenous retrovirus and LINE1 repeats, expressed in these cells [32,35,41,42].

Dppa2/4 were initially discovered as strong inducers of the 2C-like state through a candidate-based screen where oocyte/zygotic epigenetic factors were overexpressed in mESCs and the effects on ZGA-like transcription assessed [32]. This screen approach was recently extended using CRISPR-activation coupled with a single-cell transcriptomic read out, confirming Dppa2 as a potent activator of ZGA-like transcription in ESCs [41]. Mechanistically, ChIP-seq experiments revealed that Dppa2/4 bind the Dux gene, but not other 2C-like genes or repeats, regulating its expression [32,35,42]. This is independent of the SAP domain, as a germ-line enriched isoform of Dppa4 lacking this domain was still able to bind the Dux gene, albeit less strongly [42]. Dux encodes a transcription factor expressed during the minor wave of ZGA which directly regulates a suite of major wave transcripts including Zscan4 and MERVL [43–45]. Dppa2/4 are expressed in all ESCs, not just the 2C-like population, which raises the question why not all ESCs express these ZGA-like transcripts. However, when viewed through the lens of an epigenetic priming factor, their presence on the chromatin may be required to keep the Dux locus open and amenable to future activation upon an additional signal in just a subset of cells.

It has yet to be formally shown whether Dppa2/4 are required to prime ZGA *in vivo* as maternal stores may be masking early embryonic phenotypes of the reported zygotic knockout mice. While most Dppa2/4 zygotic knockout mice die shortly after birth, surviving null animals have reduced fertility, particularly when paired with another null animal [29,30], although whether this is due to ZGA failure or later developmental defects remains to be determined. While we wait for thorough characterisation of maternal Dppa2/4 knockouts, several lines of evidence support a role in fine-tuning priming of ZGA genes *in vivo.* Injection of a putative dominant negative form of Dppa2 lacking the SAP domain in zygotes led to 2-cell arrest [46] suggesting that a functional Dppa2/4 heterodimer may be required. However, too much Dppa2/4 may be just as detrimental as not enough. Zygotic injection of mutant Dppa2 that is unable to be SUMOylated impaired embryonic development, with insufficient up-regulation of ZGA transcripts at the two-cell stage [35]. It is unclear whether this mutant also acts as a dominant negative by sequestering functional Dppa2/4 from its target sites. Further maternal knockout mouse models will be important to tease apart the precise role of Dppa2/4 in ZGA *in vivo*, keeping in mind that phenotypes may be subtle due to the very nature of epigenetic priming factors.

### Roles in gastrulation: Dppa2/4 prime bivalent chromatin

Dppa2/4 have recently been shown to have an additional important role in priming the epigenome in pluripotent cells, facilitating cell differentiation by regulating bivalent chromatin [20,47]. This is achieved by regulating levels of H3K4me3 and H3K27me3 at these promoters which in turn prevents repressive DNA methylation from accumulating. Deleting either or both Dppa2/4 leads to focal losses of H3K4me3 and H3K27me3 at some but not all bivalent promoters, and a corresponding accumulation of DNA methylation [20,47]. The epigenetic changes are reversible as reintroduction of Dppa2/4 into depleted cells restores the epigenetic landscape at these genes [20]. While the precise details of how Dppa2/4 function mechanistically at these genomic loci remains to be determined, one possibility is that Dppa2/4 are involved in recruiting and/or stabilising the COMPASS and Polycomb complexes at these loci, either directly or indirectly through modulating local chromatin structure or via antisense transcription. It will be exciting to explore these, and other models of how Dppa2/4 function in the future.

Interestingly, while Dppa2/4 bind to all bivalent promoters, not all bivalent promoters are affected and those with higher levels of H3K4me3, higher levels of basal expression and the elongating form of RNA polymerase II are unaffected by Dppa2/4 loss [20]. The low level of active transcription at these unaffected ‘Dppa2/4-independent' bivalent promoters may be able to keep these genes primed and bivalent in the absence of the initiating epigenetic factor(s). The affected ‘Dppa2/4-dependent' bivalent promoters have less active transcription and so are unable to maintain their epigenetic status in the absence of Dppa2/4. This reflects one of the features of epigenetic priming factors in that they are required for the establishment and not necessarily the maintenance of a primed state.

The consequences losing Dppa2/4 and bivalent chromatin are significant. While the pluripotency of the cells remains unaffected, Dppa2/4 knockout cells are unable to efficiently differentiate in embryoid body assays [20] or directed endoderm differentiation [47], consistent with observations in previous studies [29,34]. Thus Dppa2/4 facilitate cellular differentiation and the acquisition of new cell fates. Interestingly, knockout cells eventually differentiate, albeit at lower frequency, suggesting that priming by Dppa2/4 improves the efficiency of differentiation rather than acting as a strict gatekeeper. In this way, Dppa2/4 lower the activation threshold to initiate new transcriptional programmes at a time when the embryo body plan is still being established and cells need to quickly and dynamically respond to external cues. This may provide a molecular explanation for the phenotypes of the zygotic knockout mice [29,30] which survive embryogenesis and are able to generate all cell types of the three germ layers. It is possible that this not as efficient or delayed in Dppa2/4 knockout embryos, or that cell identities have subtle defects that are not detrimental until after birth. It will be exciting to profile Dppa2/4 mutant embryos at early embryonic time points to see if there are shifts in cell composition or differentiation delays, and link this to any epigenetic alterations in gastrulating embryos. Nonetheless, these recent studies reveal how epigenetic priming factors, such as Dppa2/4, set the stage for cellular differentiation by ensuring timely activation of key developmental genes. It is unlikely that Dppa2/4 act alone during this key developmental window. The lessons learnt from studying Dppa2/4 will aid the discovery other epigenetic priming factors and add to our understanding of how cell plasticity is regulated epigenetically during development.

### Dppa2/4 facilitate reprogramming to pluripotency

Normal development is unidirectional with cells losing potency as they acquire cell fates and the embryo gains cell complexity, however it is possible to reverse this experimentally and reprogram differentiated somatic cells to pluripotency. By inducing expression of four transcription factors (Oct4, Klf4, Sox2 and Myc), fully differentiated cells can be reprogrammed to induced pluripotent stem cells (iPSCs), however this is inefficient and often incomplete. Dppa2/4 are re-expressed in the early stages of reprogramming [48] and mark cells that are destined to become iPSCs [49]. In addition to being useful markers, Dppa2/4 also have instructive roles in iPSC reprogramming. While it is possible to generate iPSCs from Dppa4 knockout MEFS [29], the frequency and efficiency is unclear. Furthermore double Dppa2/4 knockout mouse embryonic fibroblasts (MEFs) fail to reprogram, and can only be rescued by reintroducing both Dppa2 and Dppa4 together into the cells [50]. Conversely, overexpression of Dppa2/4 greatly enhances both the speed and efficiency of iPSC reprogramming in both mouse and human cells [50]. This is achieved by remodelling the epigenetic landscape, increasing global levels of H3K4me3, H3K27ac, H3K27me3 as well as the DNA damage marker γH2A.X, and promoting an alternative reprogramming trajectory [50]. The binding profile for Dppa2/4 in MEFs is largely the same as in ESCs [50] suggesting that the forced expression of Dppa2/4 in the differentiated MEFs may facilitate its acquisition of a pluripotent epigenetic landscape, thereby improving the overall efficiency of reprogramming.

## Hijacking developmental epigenetic priming factors in cancer

While cancer is generally considered a genetic-driven disease, it is becoming more apparent that epigenetic aberrations have a major contribution to the development and progression of this disease (reviewed in [51]). The epigenetic landscape usually provides a stable cell identity while priming cells for change. Cancer cells frequently have a distorted cell identity and adapt epigenetic, transcriptomic and phenotypic properties associated with embryonic cells [52–54]. The epigenome of cancer cells often becomes hyper-plastic, with cancer cells either activating normally silent transcripts such as early embryonic genes, or failing to activate transcriptional programmes upon receiving a physiological signal (reviewed in [55]). Epigenetic priming factors, responsible for establishing this dynamic and permissive landscape, are often deregulated in cancers. For example Dppa2/4 are frequently up-regulated in human cancers including lung [56], ovarian [57], colon [58,59], colorectal [60] and gastric cancers [61], amongst others [62–66], likely due to the global demethylation that occurs in cancer cells, and are often associated with poor prognosis [57–61,65]. Currently the majority of studies have been correlative, however there are some research suggesting that Dppa2/4 may be contributing to tumorigenesis. Overexpression of Dppa2/4 in mouse 3T3 cells is able to transform the cells [63]. Furthermore, Dppa2/4 enhanced soft agar colony formation and increased tumour growth in mouse xenograft experiments [63] supporting a causative role. Future studies will be key to understanding how Dppa2/4, as well as other epigenetic priming factors, contribute to the initiation, development and metastasis of cancer.

**Figure 4.**
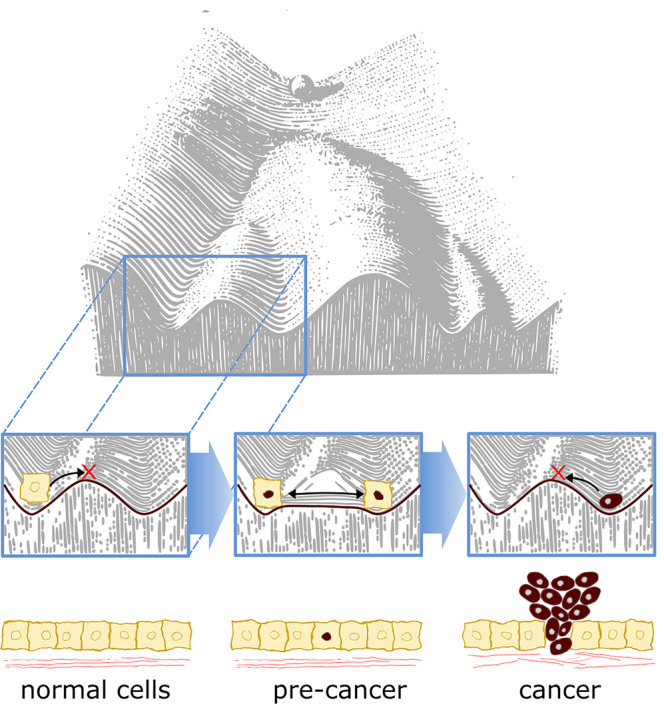
Aberrant expression of epigenetic priming factors may facilitate cancer progression. At the base of Waddington's landscape cells are normally restricted in their cell identity, unable to cross the ridges that separate them from other cell fates (left box). In precancer cells (middle box), aberrant expression of epigenetic priming factors may lead to heightened epigenetic plasticity (red nucleus), reducing the restrictions on cell identity and enabling cells to acquire new features. This may be transient as cancers lose expression of the epigenetic priming factor and become locked into their new cell identity (right box).

How could an epigenetic priming factor contribute to tumorigenesis? In 1957, Conrad Waddington postulated that development can be depicted as a ball rolling down a mountain in an epigenetic landscape, becoming progressively restricted with time as it enters valleys and is unable to cross over ridges to access other developmental endpoints (Figure 4) [67]. This restriction can be conceptualised as activation barriers, preventing differentiated cells from reverting to earlier more potent states. Epigenetic priming factors may function by lowering this epigenetic barrier, increasing the epigenetic plasticity and range of identities and phenotypes available to the cell. Importantly, this does not need to be sustained, once a cell has adopted a new identity or capability, such as increased stemness or cell motility, it can remain in that state, even if expression of the epigenetic priming factor and associated increased epigenetic plasticity is reversed (reviewed in [68]). Normally during development, the expression and activity of epigenetic priming factors is tightly controlled so that embryogenesis proceeds in an orderly and regulated manner. However, cancer cells can activate epigenetic priming factors and increase their epigenetic plasticity. By hijacking these early developmental processes, cancer cells break free of normal restrictions and control and take on new functions and identities. By increasing our understanding of how epigenetic priming factors function in normal development, we can use the principles learnt to gain new insights of their contribution to disease.

## Perspectives

- Epigenetic priming factors function by increasing the epigenetic plasticity of a cell, poising promoters or enhancers for future activation. This effectively lowers the ‘epigenetic barrier’ of the cell, enabling it to adopt new transcriptional programmes more efficiently upon receiving signals to differentiate during development, or in response to stress, injury or hormonal cues in adults.
- Dppa2/4 are exemplars of epigenetic priming factors, acting as facilitators of cell fate transitions in early development and reprogramming.
- Developmental epigenetic priming factors may be hijacked in cancers to promote cell plasticity and facilitate acquisition of new identities or functions. Mechanistic understanding of the role of epigenetic priming factors in normal development will provide insights into how they contribute to disease.

## Abbreviations

ChIP: chromatin immunoprecipitation
DNMT1: DNA methyltransferase 1
Dppa2/4: Developmental Pluripotency Associated 2 and 4
ESC: embryonic stem cell
H3K27me3: histone H3 lysine 27 trimethylation
H3K4me3: histone H3 lysine 4 trimethylation
H4K16ac: histone H4 lysine 16 acetylation
iPSC: induced pluripotent stem cell
MEFs: mouse embryonic fibroblasts
ZGA: zygotic genome activation
